# Coordinated adaptation of *Staphylococcus aureus* to calprotectin-dependent metal sequestration

**DOI:** 10.1128/mbio.01389-24

**Published:** 2024-06-26

**Authors:** Valeria M. Reyes Ruiz, Jeffrey A. Freiberg, Andy Weiss, Erin R. Green, Mary-Elizabeth Jobson, Emily Felton, Lindsey N. Shaw, Walter J. Chazin, Eric P. Skaar

**Affiliations:** 1 Department of Pathology, Microbiology, and Immunology, Vanderbilt University Medical Center, Nashville, Tennessee, USA; 2 Vanderbilt Institute for Infection, Immunology, and Inflammation, Vanderbilt University Medical Center, Nashville, Tennessee, USA; 3 Division of Infectious Diseases, Department of Medicine, Vanderbilt University Medical Center, Nashville, Tennessee, USA; 4 Department of Cell Biology, Microbiology, and Molecular Biology, University of South Florida, Tampa, Florida, USA; 5 Department of Biochemistry, Vanderbilt University, Nashville, Tennessee, USA; Universite de Geneve, Geneva, Switzerland

**Keywords:** *Staphylococcus aureus*, calprotectin, nutritional immunity, clpP

## Abstract

The host protein calprotectin inhibits the growth of a variety of bacterial pathogens through metal sequestration in a process known as “nutritional immunity.” *Staphylococcus aureus* growth is inhibited by calprotectin *in vitro*, and calprotectin is localized *in vivo* to staphylococcal abscesses during infection. However, the staphylococcal adaptations that provide defense against nutritional immunity and the role of metal-responsive regulators are not fully characterized. In this work, we define the transcriptional response of *S. aureus* and the role of the metal-responsive regulators, Zur, Fur, and MntR, in response to metal limitation by calprotectin exposure. Additionally, we identified genes affecting the fitness of *S. aureus* during metal limitation through a Transposon sequencing (Tn-seq) approach. Loss of function mutations in *clpP*, which encodes a proteolytic subunit of the ATP-dependent Clp protease, demonstrate reduced fitness of *S. aureus* to the presence of calprotectin. ClpP contributes to pathogenesis *in vivo* in a calprotectin-dependent manner. These studies establish a critical role for ClpP to combat metal limitation by calprotectin and reveal the genes required for *S. aureus* to outcompete the host for metals.

**IMPORTANCE:**

*Staphylococcus aureus* is a leading cause of skin and soft tissue infections, bloodstream infections, and endocarditis. Antibiotic treatment failures during *S. aureus* infections are increasingly prevalent, highlighting the need for novel antimicrobial agents. Metal chelator-based therapeutics have tremendous potential as antimicrobials due to the strict requirement for nutrient metals exhibited by bacterial pathogens. The high-affinity transition metal-binding properties of calprotectin represents a potential therapeutic strategy that functions through metal chelation. Our studies provide a foundation to define mechanisms by which *S. aureus* combats nutritional immunity and may be useful for the development of novel therapeutics to counter the ability of *S. aureus* to survive in a metal-limited environment.

## INTRODUCTION

*Staphylococcus aureus* is associated with more than one million deaths per year (1). It is a leading cause of skin and soft tissue infections, endocarditis, and bloodstream infections (2, 3). Despite the availability of suitable antimicrobial therapy, *S. aureus* infections have high rates of treatment failure. Bacterial pathogens require transition metals for the catalytic function of enzymes, stabilization of protein structure, and regulation of gene expression (4, 5). During infection, *S. aureus* must acquire essential nutrient metals from the vertebrate host to survive and replicate. In turn, the mammalian host sequesters metals to restrict the growth of bacteria through processes known as “nutritional immunity” (6). Vertebrate immune cells engage in nutritional immunity by secreting metal-chelating innate immune proteins such as calprotectin (CP). CP is a heterodimer of the S100A8 and S100A9 proteins that chelates multiple nutrient metals. S100A8 and S100A9 contain two distinct metal-binding sites at the dimer interface: a canonical His3Asp site and a unique His6 site. The His6 site possesses broad metal-binding capabilities with a high affinity for zinc (Zn), copper (Cu), manganese (Mn), iron (Fe), and nickel (Ni) (7–10). The His3Asp site binds Zn and Cu with high affinity in the picomolar to nanomolar range (7, 11, 12).

Calprotectin inhibits *S. aureus* growth *in vitro* and is localized *in vivo* to staphylococcal abscesses (7, 13, 14). CP accounts for 40% to 50% of the total protein content in neutrophils, which are rapidly recruited to the site of infection (15). The contribution of CP to host defense against *S. aureus* infection varies across *in vivo* models, and CP distribution within staphylococcal abscesses differs between infected organs. For example, CP is found throughout staphylococcal abscesses in the liver and kidney, whereas in the heart, CP is only present surrounding staphylococcal abscesses and is absent from the abscess center (14, 16, 17). Thus, the exact mechanisms by which *S. aureus* mitigates CP-dependent nutritional immunity are not fully understood.

Intracellular levels of transition metals are sensed by transcriptional regulators, allowing bacteria to coordinate their response to metal starvation. In *S. aureus*, the major metal-sensing regulators include Fur, Zur, and MntR, which bind and respond to Fe, Zn, and Mn, respectively (6, 18, 19). These regulators bind their cognate metal and dimerize, typically repressing the expression of genes involved in metal homeostasis. In a metal-deplete environment, Fur, Zur, and MntR no longer bind to the promoters of their regulon, which allows for de-repression of genes encoding metal import systems, metallochaperones, siderophore synthesis, and other targets (18–22).

Here, we aimed to understand how *S. aureus* adapts to multimetal nutrient limitation by CP. Leveraging omics approaches including RNA sequencing (RNA-seq) and transposon sequencing (Tn-seq), we generate a powerful data set to better understand the adaptation of *S. aureus* to metal starvation imposed by CP and the role of Fur, Zur, and MntR in this response. In addition to genes that have previously been shown to aid *S. aureus* in response to disruptions in metal homeostasis, we identified previously uncharacterized genes whose expression is altered or that affect the fitness of *S. aureus* in response to CP. Our studies also describe the transcriptional changes in small RNAs in response to metal limitation. Finally, we report a role for the proteolytic subunit of the Clp protease system, ClpP, in protecting *S. aureus* from CP-mediated stress using a systemic model of infection.

## RESULTS

### *S. aureus* transcriptional responses to calprotectin exposure

To study the response of *S. aureus* to nutrient metal limitation by treatment with CP, a global transcriptomic analysis by RNA-seq was performed on wild-type (WT) *S. aureus* strain grown in media with either WT calprotectin (WTCP), which restricts bacteria from Zn, Fe, Mn, Cu, and Ni, or a His6 site mutant calprotectin (∆s1CP), which only restricts Zn and Cu (7–12). The concentrations of calprotectin that reduced growth without completely restricting growth were determined by growing WT *S. aureus* with increasing concentrations of CP compared with a vehicle control (Fig. S1A). Based on these experiments, a concentration of 300 µg/mL WT CP was chosen. As the ∆s1CP mutant has half the capacity to bind metals, double the concentration of ∆s1CP (600 µg/mL) was added for an equivalent total number of metal binding sites (Fig. S1B). Samples were collected at the mid-exponential phase for all three conditions (WTCP, ∆s1CP, and vehicle control).

**Fig 1:**
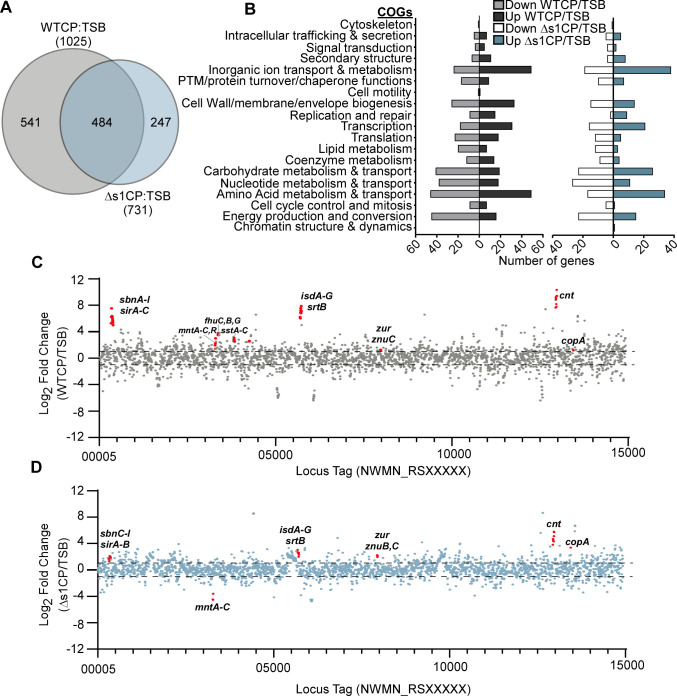
Genes that are differentially expressed in response to calprotectin. RNA sequencing was performed upon treatment with calprotectin (WTCP) or site 1 mutant calprotectin (∆s1CP) compared with vehicle control [TSB + calprotectin buffer (TSB)]. The Log_2_ fold change cutoff is ≥1 in each direction, and the *P*-value cutoff is <0.05. (**A**) Venn diagram of differentially expressed genes in response to calprotectin and ∆s1CP. (**B**) Genes identified in the RNA-seq analysis with significantly differential expression grouped into Clusters of Orthologous Genes (COGs). (**C**) Log_2_ fold change in WTCP (**D**) or ∆s1CP treatment compared with vehicle control and represented across the genome of *S. aureus*. Dashed line represents a Log_2_ fold change of 1 or −1. Genes highlighted have been previously described as participating in metal homeostasis.

Compared with the vehicle control condition, treatment with WTCP resulted in the differential expression of 1025 genes with greater than 2-fold change in expression, 504 genes upregulated, and 521 genes downregulated (Fig. 1A; Table S1). By contrast, treatment with ∆s1CP caused differential expression of 731 genes compared with the vehicle control, with 426 genes upregulated and 305 genes downregulated (Fig. 1A; Table S2). There were 484 genes differentially expressed in both WTCP and ∆s1CP treatments. To analyze the functional consequences of CP treatment, EggNOG-Mapper was used to annotate each differentially expressed gene with a Cluster of Orthologous Group (COG) (23, 24). The COG most represented in the upregulated genes upon treatment with WTCP and ∆s1CP was “inorganic ion transport and metabolism.” There was also an enriched representation of genes involved in amino acid metabolism and transport. Additionally, there were changes in the expression of genes involved in “cell wall, membrane, and envelope biogenesis.” The COG that was primarily represented by the downregulated genes was “energy production and conversion” (Fig. 1B). These results suggest that the response of *S. aureus* to nutrient metal limitation by CP yields changes in core cellular processes.

Among genes whose expression was significantly upregulated in response to WTCP was the *sbn* locus that encodes for enzymes important for the synthesis of the siderophore staphyloferrin B, and the *isd* system, which is important for heme-iron uptake (25–29). Expression was increased for multiple systems important for the acquisition of siderophores and for combatting iron limitation, including the *sirA-C* operon, which is required for binding and translocation of staphyloferrin B, the ferrichrome uptake operon (*fhu*), and the *sstABCD* operon that allows *S. aureus* to utilize catechol siderophores (30–33). The genes encoding the Mn transporter, *mntABC*, and its transcriptional regulator *mntR*, increased only after treatment with WTCP as is expected since ∆s1CP does not bind Mn with high affinity (34). Consistent with this, expression of the *cnt* operon, which encodes for the metallopore staphylopine and provides *S. aureus* with the ability to compete with calprotectin for Zn was increased with both WTCP and ∆s1CP treatments (35). Genes encoding for the components of the Zn import system, *znuABC*, also had increased expression in both conditions (35). Transcripts for *copA*, a transmembrane copper (Cu) exporter, were increased upon treatment with both WTCP and ∆s1CP (Fig. 1C and D) (36). Together, these RNA-seq data revealed that the transcriptome of *S. aureus* changes drastically upon nutrient metal starvation with WTCP and ∆s1CP.

### Role of metal-dependent regulators in coordinating the response to calprotectin treatment

*S. aureus* utilizes metallo-sensing DNA-binding transcriptional regulators (Zur, Fur, and MntR) to mediate transcriptional changes that aid in counteracting disruptions in metal homeostasis (6, 18, 19). To define the role of metal-sensing regulators Fur, Zur, and MntR in the transcriptional response of *S. aureus* to CP exposure, RNA-seq was performed on the respective mutant strains compared with WT *S. aureus*. Considering the canonical function of Fur, Zur, and MntR as repressors under replete metal conditions, their regulons were determined by comparing genes differentially expressed in WT *S. aureus* to a regulator mutant in vehicle control conditions that were replete with metals. A shortcoming of this approach is that the data set excludes genes that do not follow the canonical regulatory activity of Fur, Zur, and MntR acting as repressors. The differentially expressed genes in vehicle control between WT or Fur, Zur, and MntR mutants (Tables S3 to S5) were then compared with the list of genes differentially expressed upon calprotectin treatment in WT *S. aureus* (Tables S1 and S2).

**Fig 2:**
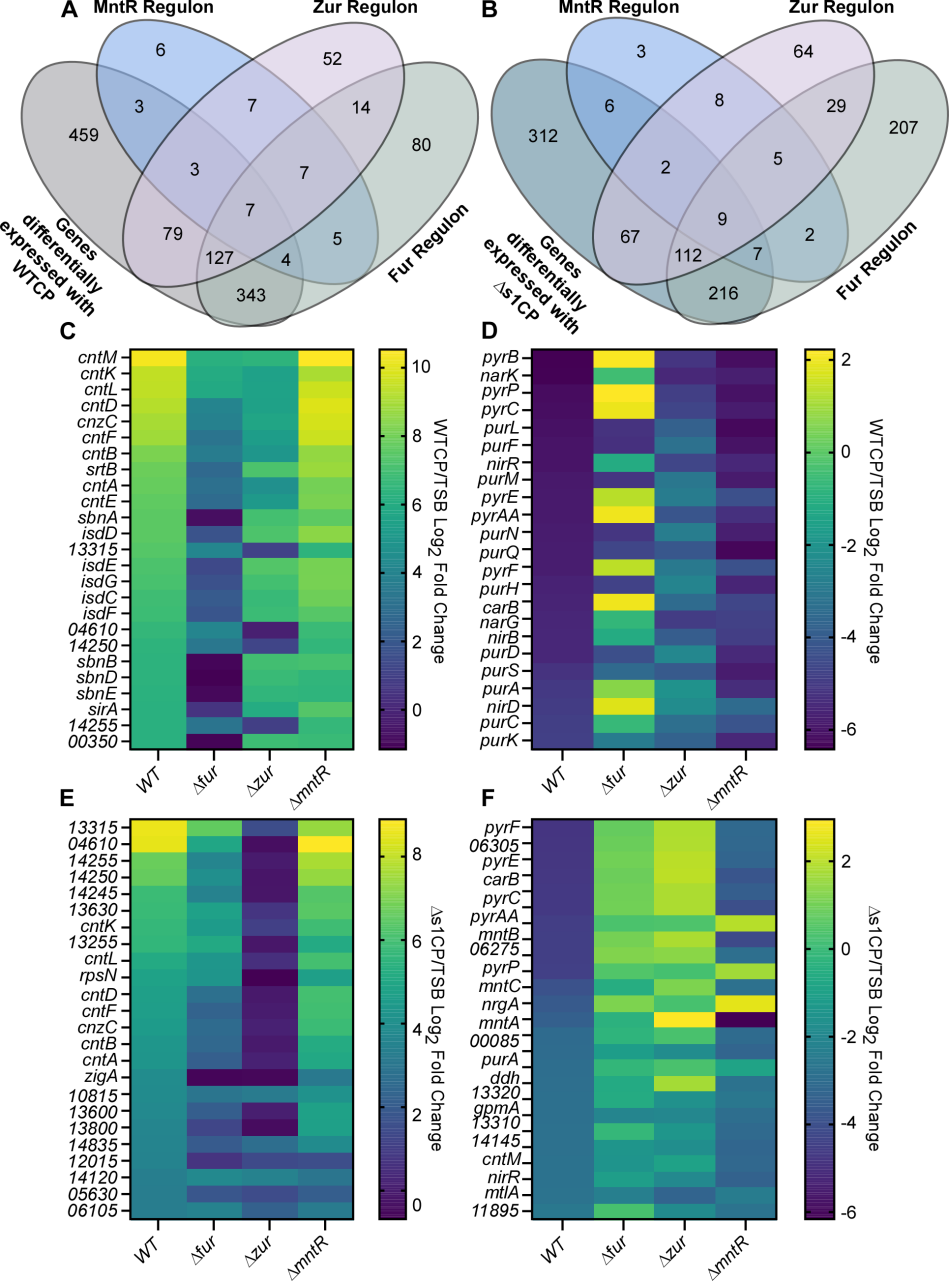
Role of metal-dependent regulators in coordinating the response to metal starvation by calprotectin. The regulons of Fur, Zur, and MntR were determined under the assumption that they are acting as repressors. RNA-seq data from WT *S. aureus* and each regulator mutant (∆*fur*, ∆*zur*, or ∆*mntR*) were compared with the vehicle control. Genes that are differentially expressed in the mutant strain compared with WT were identified as part of the regulon. These genes were then compared with the genes that were differentially expressed in response to (**A**) WTCP (**B**) or ∆s1CP as described in Fig. 1, and overlap is represented in a Venn Diagram. Heat maps were generated for the top 25 genes with either the greatest (**C**) increase in expression upon WTCP treatment, (**D**) decrease in expression upon WTCP treatment, (**E**) increase in expression upon ∆s1CP treatment, or (**F**) decrease in expression upon ∆s1CP treatment. The value of each regulator mutant strain in response to WTCP or ∆s1CP compared with vehicle control is also represented on the heat maps.

There are 17 genes differentially expressed in the WTCP condition that are also part of the MntR regulon, 216 genes changed upon treatment with WTCP are part of the Zur regulon, and 481 genes changed upon treatment with WTCP are part of the Fur regulon (Fig. 2A). Upon treatment with ∆s1CP, 24 differentially expressed genes are part of the MntR regulon, 190 genes are part of the Zur regulon, and 344 genes are part of the Fur regulon (Fig. 2B). Fur and Zur are the main regulators for the top 50 genes that are differentially expressed upon treatment with WTCP in WT *S. aureus* (Fig. 2C and D). In contrast to treatment with WTCP, Zur appears to be the predominant regulator for the top 50 genes differentially expressed upon treatment with ∆s1CP (Fig. 2E and F). This is consistent with ∆s1CP only having one metal binding site that binds Zn and Cu (10–12).

Our analysis revealed a total of 459 genes differentially expressed upon WTCP exposure and 312 genes differentially expressed with ∆s1CP treatment that do not appear to be canonically regulated by Fur, Zur, or MntR (Tables S6 and S7). These genes may be affected through alterations in the homeostasis of other metals including Cu and Ni. Additionally, these genes may be regulated by additional transcription factors that respond to cellular metal concentrations or may not follow the canonical regulation by Fur, Zur, and MntR acting as repressors. Further analysis is needed to determine whether these genes have an impact on the adaptation of *S. aureus* to metal starvation. Collectively, these results suggest that Fur, Zur, and MntR play distinct roles in the *S. aureus* response to nutrient metal limitation.

### Multiple small RNAs are differentially expressed in response to calprotectin

**Fig 3:**
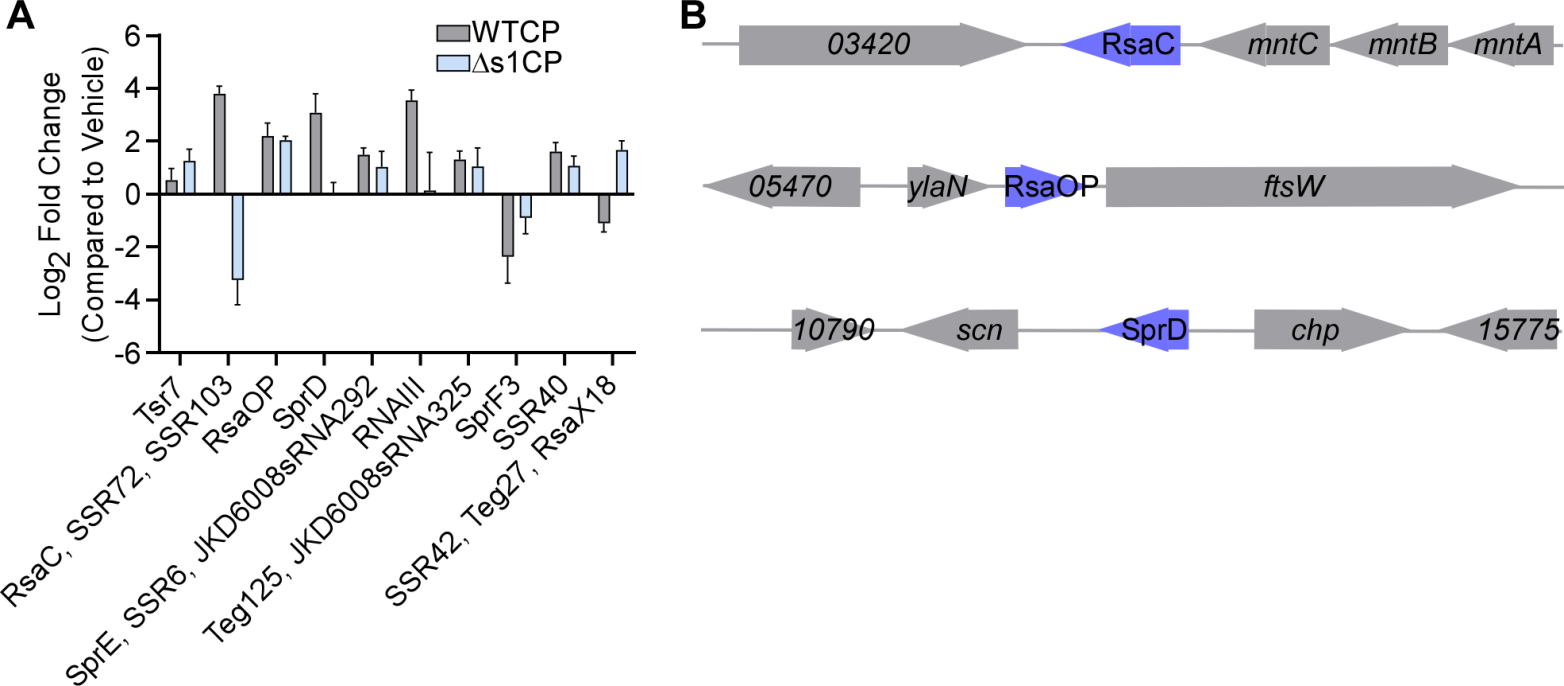
Small RNAs are differentially expressed in response to calprotectin. The frequency of known sRNAs in each condition was identified using a minimum cutoff of 100 in called frequency and Log_2_ fold change of 1 or −1. (**A**) Frequencies for each sRNA were then used to calculate the Log_2_ fold change comparing WTCP or ∆s1CP treatment with vehicle control. (**B**) Three of the differentially expressed sRNAs upon CP treatment are highlighted with the surrounding genes. To better highlight the sRNAs with a range in sizes, the three genomic regions are at scale within themselves, but they are not comparable with each other.

Small RNAs (sRNAs) are important regulators of many processes during *S. aureus* infection, including metabolism, virulence, and resistance to multiple environmental stressors (37–42). The changes in sRNAs upon metal starvation are not well characterized. Thus, the RNA-seq data set was analyzed for the presence and changes of known sRNAs. The frequency of sRNAs in each condition was determined and high confidence hits were only reported if they reached a cutoff of 100 frequency in all conditions (Vehicle, WTCP, and ∆s1CP). A shortcoming of this approach is that sRNAs in the untreated sample that do not reach that cutoff were not considered for further comparisons, including sRNAs such as IsrR, which is repressed by Fur (43). After identifying the presence of sRNAs that satisfied these cutoff criteria, the fold change was calculated by comparing WTCP or ∆s1CP treatment against the vehicle control. Treatment with WTCP resulted in the identification of 9 sRNAs with greater than 2-fold change in expression, 7 sRNAs upregulated, and 2 sRNAs downregulated (Fig. 3A). By contrast, treatment with ∆s1CP resulted in 6 sRNAs upregulated and 1 sRNA downregulated (Fig. 3A). The sRNA RsaC is produced during manganese starvation and targets *sodA*, a Mn requiring enzyme (44). This is consistent with RsaC being upregulated specifically when Mn is limited during WTCP treatment. The sRNA RsaOP exhibits increased expression after treatment with WTCP and ∆s1CP and is in close proximity to the gene encoding for YlaN. Recent studies suggest a role for YlaN as an iron-binding protein that influences Fur-dependent regulation during iron starvation (45). SprD is only upregulated in response to WTCP, and it is in close proximity to the gene encoding for chemotaxis inhibitory protein (*chp*), which is also upregulated in response to WTCP. These results highlight the multiple levels of regulation employed by *S. aureus* to coordinate the response to metal starvation.

### Genes that affect the fitness of *S. aureus* upon nutrient metal limitation by calprotectin

**Fig 4:**
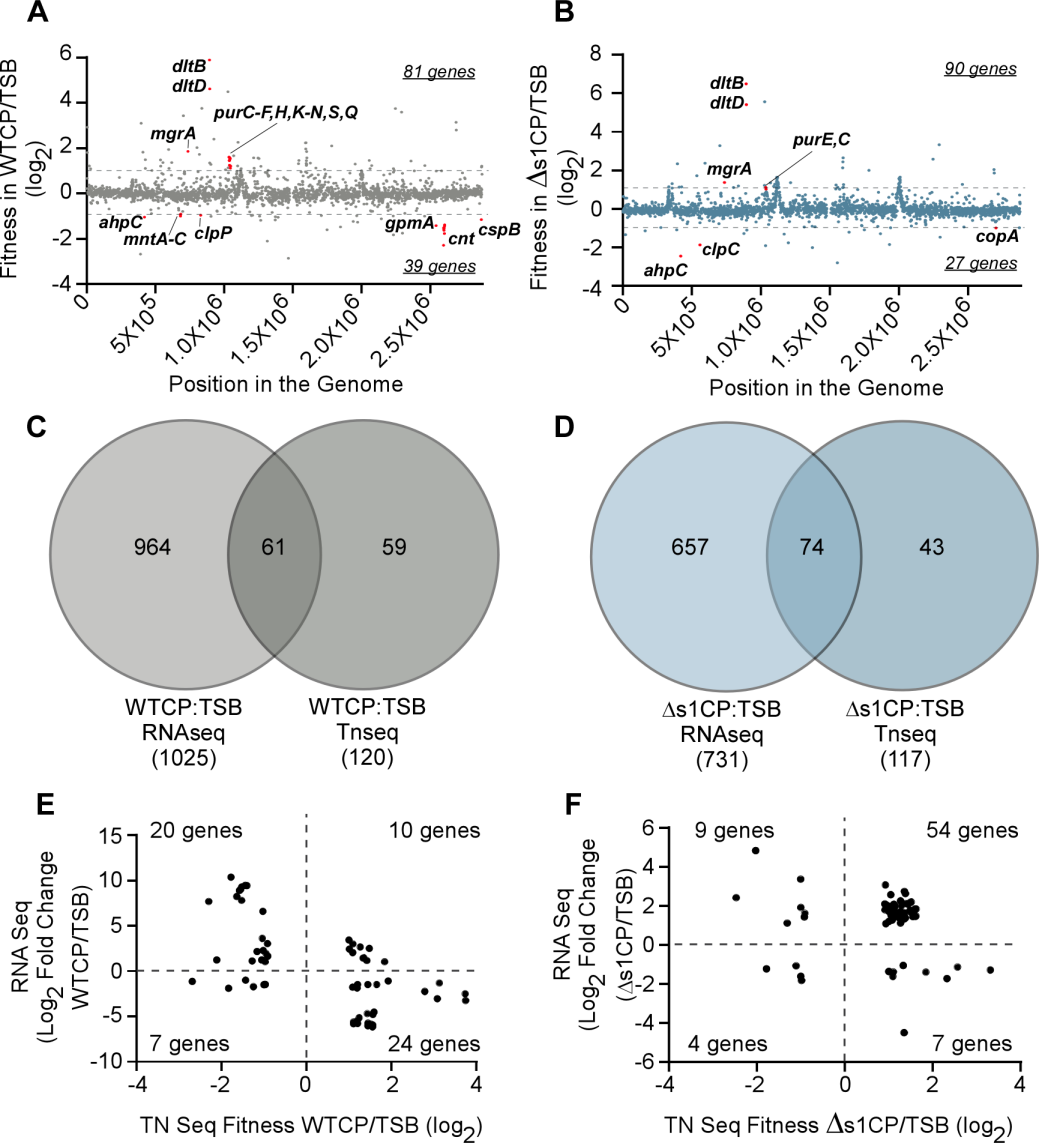
Genes that affect the fitness of *S. aureus* upon metal limitation by calprotectin. Representation of Tn-seq results for (**A**) WTCP or (**B**) ∆s1CP across the position in the genome. Z score was calculated, and a cutoff for significance was set at two standard deviations away from the mean. The dashed line represents the Log_2_ fitness value for the gene at the cutoff. Highlighted genes discussed in the text are in red. (**C, D**) Hits from the Tn-seq data set and the RNA-seq data set were compared to determine the overlap in a Venn Diagram. (**E, F**) Hits from the Tn-seq data set and the RNA-seq that overlapped with corresponding Log_2_ fold change in RNA-seq or Log_2_ fitness value in Tn-seq for (**E**) WTCP or (**F**) ∆s1CP.

In addition to examining changes in gene expression, these studies sought to uncover genes that affect the fitness of *S. aureus* when the bacteria experiences calprotectin-mediated metal starvation by performing a genome-wide Tn-seq screen on bacteria grown in the presence of WTCP or ∆s1CP. First, a transposon library was created and confirmed to have approximately 80,000 independent transposon mutants as verified by Illumina sequencing analysis. Nearly 31% of all TA sites and at least one TA site in 93.5% of annotated open reading frames in the Newman genome (2,666 out of 2852) had transposon insertions. Exposure to WTCP reduced the fitness of 39 transposon mutants and increased the fitness of 81 mutants relative to vehicle control (Fig. 4A; Table S8). By contrast, exposure to ∆s1CP resulted in the reduced fitness of 27 transposon mutants and increased the fitness of 90 transposon mutants (Fig. 4B; Table S9). Mutants harboring transposon insertions in *dltD* and *dltB* were the most enriched following treatment with both WTCP and ∆s1CP, compared with vehicle-treated control cultures (Fig. 4A and B). The *dlt* operon catalyzes the introduction of D-alanine into teichoic acids (46). Transposon mutants with disruptions in *de novo* purine biosynthesis pathways also had increased fitness. Additionally, mutants harboring transposon insertions in *mgrA* had increased fitness in response to both WTCP and ∆s1CP (Fig. 4A and B). By contrast, mutants with transposon insertions in *mntABC*, *gpmA*, and the *cnt* operon had decreased fitness only when exposed to WTCP (Fig. 4A). These findings validate our approach as this is consistent with: (1) MntABC being the primary transporter responsible for Mn uptake in the presence of calprotectin, (2) the Cnt system allowing for staphylopine-mediated metal acquisition, and (3) GpmA enabling *S. aureus* to maintain the ability to consume glucose in Mn-starved conditions (17, 47). Mutants with transposon insertions in the gene encoding the alkyl hydroperoxide reductase, AhpC, were more sensitive to metal starvation by both WTCP and ∆s1CP (Fig. 4A and B). In addition, mutants with transposon insertions in genes related to copper homeostasis were selected against when treated with calprotectin such as the *copA* mutant, which was selected against with ∆s1CP (Fig. 4A and B). Transposon insertions in genes encoding for the Clp protease system had reduced fitness in treatments with both WTCP and ∆s1CP.

When comparing the RNA-seq data set with the Tn-seq data set, 61 genes for WTCP and 74 genes for ∆s1CP are shared, showing both differential expression and an impact on fitness (Fig. 4C and D, Tables S10 and S11). These included genes encoding metal importers and acquisition systems, which are upregulated in response to metal starvation and are required for optimal growth (17, 35). In response to WTCP, 20 genes followed this pattern of increased expression and decreased fitness when mutated. These include the Zn and Mn acquisition systems, *cnt* and *mntABC*, along with the alkyl hydroperoxide reductase subunit C (*ahpC*), a pyridine nucleotide-disulfide oxidoreductase (*NWMN_RS04610*), a putative DNA binding protein (*NWMN_ RS06710*), and hypothetical proteins such as NWMN_RS05475 and NWMN_RS10060 (Table S10). In response to WTCP, 24 genes with decreased expression showed an increased bacterial fitness when mutated (Fig. 4E; Table S10). These included *de novo* purine biosynthesis pathway genes with decreased expression in the RNA-seq analysis and increased fitness when the genes had transposon insertions. A few genes had increased expression of the transcript with increased bacterial fitness when mutated (10 genes) or decreased expression of the transcript with decreased bacterial fitness when mutated (7 genes). Some of these include *clpP*, *gpmA*, and multiple genes with unknown functions (Fig. 4E; Table S8). When comparing genes upon treatment of ∆s1CP that were hit in both the RNA-seq and Tn-seq data sets, many had increased expression of the associated transcript and increased bacterial fitness when mutated in response to ∆s1CP compared with the vehicle control (54 genes). The majority of these genes are hypothetical or annotated to encode phage proteins (Fig. 4F; Table S11). Taken together, these results reveal novel genes that play a role in *S. aureus* adaptation to CP-mediated nutrient metal starvation.

### ClpP promotes *S. aureus* response to calprotectin-mediated nutrient metal starvation

**Fig 5:**
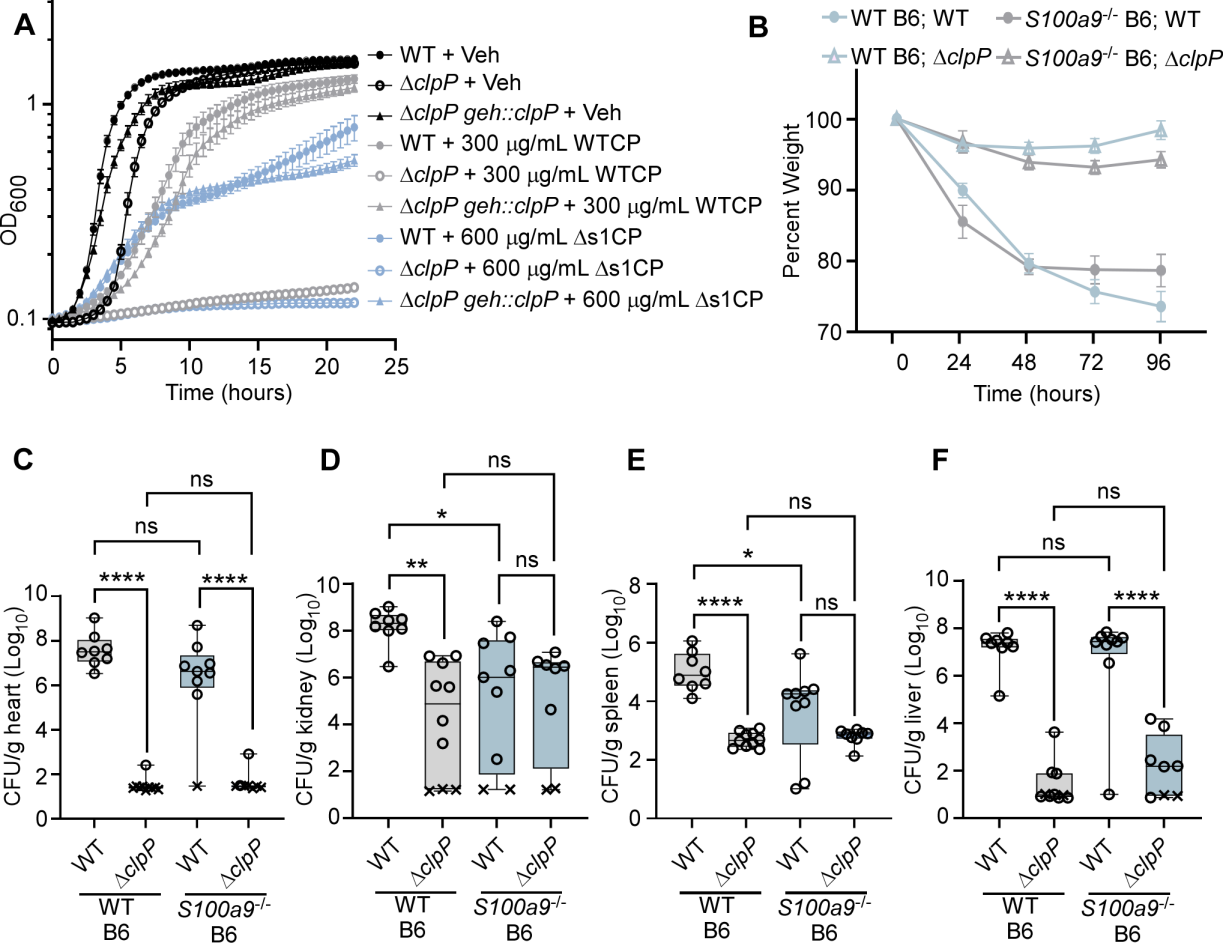
ClpP is required to overcome CP-dependent metal limitation. (**A**) Growth kinetics of *S. aureus* WT, ∆*clpP*, or a complemented strain (∆*clpP geh::clpP*) in vehicle, 300 µg/mL WTCP, or 600 µg/mL ∆s1CP were monitored for 24 h. Data shown are averages of three independent experiments (mean ± SEM). (**B–F**) *S. aureus* WT or ∆*clpP* were systemically inoculated in WT or S100A9-deficient mice (*S100a9^−^*^/−^), and weights were monitored for 96 h. (**B**) Percent weight is shown relative to the weight before infection. CFUs recovered at 96 h post-infection in the (**C**) heart, (**D**) kidney, (**E**) spleen, and (**F**) liver. (**C–F**) Statistical analysis is done with two-way ANOVA with Tukey’s multiple comparison post-test on log_10_-transformed data. *P < 0.05, **P < 0.01, ***P < 0.001, ****P < 0.0001, ns = not significant. X symbols represent the limit of detection. Graphs include data from two individual experiments with 8 to 10 mice per group.

The RNA-seq and Tn-seq data sets allowed us to identify novel factors aiding in the adaptation of *S. aureus* in response to CP. To expand on this knowledge, the role of one of the hits, *clpP*, was tested both in culture and during infection. The Clp protease regulates many physiological processes, including metabolism and virulence (48). Our studies previously demonstrated that *clp* genes impact heme acquisition (49). In this work, mutants with disruptions in *clpP* and *clpC* had decreased fitness in response to WTCP or ∆s1CP, respectively (Fig. 4A and B). These data suggest that the Clp system has a role in the response to metal starvation beyond iron limitation, as the ∆s1CP mainly restricts Zn. To better understand the role of ClpP in response to metal starvation, growth assays in the presence of calprotectin were performed using WT *S. aureus* and a ClpP-deficient strain of *S. aureus* (∆*clpP*). The ∆*clpP* strain showed decreased growth, compared with WT *S. aureus,* in response to both WTCP and ∆s1CP (Fig. 5A). This growth defect in ∆*clpP* upon treatment with WTCP or ∆s1CP can be complemented by the expression of *clpP in trans* (Fig. 5A). We evaluated the impact of WT calprotectin on metal levels in WT and ∆*clpP* and found no significant difference in Mn, Zn, or Fe between these two strains (data not shown). This finding suggests that the growth defect of a ∆*clpP* is not due exclusively to decreased metal uptake and that ClpP supports the ability of *S. aureus* to combat metal limitation by calprotectin.

To test whether the Clp system contributes to combating metal starvation during infection, C57BL/6J (WT) or *S100a9*^−/−^ mice, which are deficient for calprotectin, were infected via a retroorbital injection with either WT or ∆*clpP S. aureus*. Mice infected with WT *S. aureus* showed significant weight loss over the course of 96 h compared with the mice infected with ∆*clpP*, and this was independent of the mouse genotype (Fig. 5B). WT mice infected with ∆*clpP* resulted in reduced bacterial burdens in the heart, kidney, spleen, and liver compared with mice infected with WT *S. aureus* (Fig. 5C through F). However, no differences were observed when comparing bacterial burdens of ∆*clpP* and WT *S. aureus* in the kidney and spleen of *S100a9*^−/−^ mice (Fig. 5C through F). These data suggest that ClpP is important in the pathogenesis of *S. aureus in vivo* and partially dependent on nutrient metal starvation by calprotectin. ClpP plays a role in combating calprotectin-mediated nutrient metal starvation specifically in the kidney and spleen, whereas it may play different roles in the pathogenesis of *S. aureus* in the heart and liver. Taken together, these results revealed factors that enable *S. aureus* to combat calprotectin-dependent metal starvation during infection.

## DISCUSSION

The battle for essential metals between the mammalian host and bacterial pathogens is critical for disease outcomes (6). We hypothesized that *S. aureus* utilizes various adaptations mediated by metal-dependent regulators Zur, Fur, and MntR to defend against metal deficiency. This study revealed the contribution of Fur, Zur, and MntR to changes in the transcriptome of *S. aureus* in the response to nutrient metal starvation. Additionally, the genes affecting the fitness of *S. aureus* when combating nutrient metal limitation were identified revealing that *clpP,* which encodes an ATP-dependent Clp protease proteolytic subunit, contributes to the pathogenesis of *S. aureus in vivo* in a manner that is dependent on the presence of calprotectin. These studies provide a better understanding of the battle for nutrient metals between *S. aureus* and the host protein calprotectin.

Transcriptomic data revealed that for the 50 most differentially expressed genes, Fur and Zur appear to be the dominant regulators coordinating this response. In *Bacillus subtilis*, Fur coordinates the response to iron limitation in a sequential manner, where uptake systems are first upregulated, then genes encoding for siderophore production, and finally, an iron-sparing response is induced (50). Our results showed that genes involved in all of these processes were differentially expressed in the RNA-seq data set, including upregulation of the *sbn* system, *isd* system, *fhu*, *sirA-C*, and *sstABCD,* as well as downregulation of nitrate-related enzymes (43). Upon treatment with ∆s1CP, which specifically limits access to Zn, Zur appears to be the dominant regulator with the highest number of differentially expressed genes. In addition, this study identified 10 sRNAs with known annotations that were differentially expressed upon metal starvation. One of these included RsaC, which is co-transcribed with the Mn uptake system, MntABC, and produced in response to Mn starvation. RsaC targets the *sodA* mRNA, which encodes for a Mn-dependent superoxide dismutase (44). Additionally, RsaOP is in close proximity to *ylaN,* which responds to iron starvation (45). Further studies are needed to identify if *ylaN* is a direct target or if RsaOP is co-transcribed with *ylaN*. SprD RNA enhances the virulence of *S. aureus in vivo* (51) and was also upregulated in response to WTCP treatment. Identification of their targets will shed insight into the mechanism by which these sRNAs aid *S. aureus* in the response to calprotectin.

This study also identified genes affecting fitness of *S. aureus* in response to calprotectin. Mutants harboring transposon insertions in the *dlt* operon had increased fitness in response to both WTCP and ∆s1CP treatment. Dlt mediates positively charged D-alanine incorporation into teichoic acids and strains of *S. aureus* deficient for *dlt* are more sensitive to killing by human neutrophils, are attenuated in mice, and have increased sensitivity to vancomycin and other autolytic enzymes (52–54). However, this study shows an advantage for strains lacking *dlt* when *S. aureus* is exposed to CP treatment. Although the mechanism for this has yet to be characterized, potential models include that the positive charge provided by D-alanine can repel metals or that these changes to the membrane affect the incorporation of metal uptake systems into the cell envelope. The Tn-seq results also show that genes involved in Cu homeostasis impact the fitness of *S. aureus*. The Cu-exporting P-type ATPase A (CopA) is required for the export of excess Cu from the cell (36). Transposon insertions in *copA* caused decreased fitness in response to ∆s1CP. There is precedent for other organisms also having cross-talk between Zn and Cu homeostasis. For example, extracellular Zn stress induces Cu depletion in *Acinetobacter baumannii* (55), and in organisms such as *Streptococcus agalactiae,* there is cross-talk between Cu and Zn efflux systems (56). A potential model for CopA to provide an advantage in a Zn-limited environment is that Zn depletion may cause toxic levels of Cu inside the bacterium, thus requiring a Cu efflux system to enable growth. This was recently demonstrated in *S. aureus* where the usage of Zn acquisition systems facilitates bacterial Cu uptake and a response for detoxification of Cu (57).

The Clp protease alters the abundance of Isd, a heme-iron acquisition system (49). This study showed that mutations in genes encoding components of the Clp system exhibited reduced fitness following exposure to both WTCP and Δs1CP. This suggests that the role of Clp in response to nutrient metal starvation expands beyond combatting Fe restriction, as Δs1CP predominantly mediates Zn limitation. *S. aureus* deficient in *clpP* showed decreased growth *in vitro* when exposed to calprotectin and reduced pathogenesis *in vivo,* which was dependent on the presence of calprotectin. In a previous study, we performed proteomic analysis of Δ*clpP* compared with WT *S. aureus* in response to low Fe conditions, which revealed 84 proteins with altered abundance (58). Proteins with altered abundance included GpmA, which is decreased in Δ*clpP* compared with WT *S. aureus* when treated with an Fe chelator. *S. aureus* has two variants of phosphoglycerate mutase, GpmI, which is Mn-dependent, and GpmA, which aids in glucose consumption when Mn is limited and is Mn-independent (47). Therefore, it is possible that the Clp system regulates the abundance of multiple metabolic enzymes that are more efficient at performing their function when nutrient metals are limited. The Clp protease has been targeted by acyldepsipeptides, a class of antibiotic, and represents a potential therapeutic for other pathogens (59). Thus, further studies are important to better understand the mechanism by which the Clp system aids *S. aureus* in response to nutritional immunity.

When comparing the RNA-seq and Tn-seq data sets in response to calprotectin, there are many genes that were only affected in one dataset, which speaks to the value of these complementary approaches to identify novel factors coordinating the adaptation of *S. aureus* to nutrient metal starvation. Additionally, many hypothetical proteins are present in both data sets, showing altered expression and impacts on bacterial fitness. Thus, the results from these studies provide a rich framework for the discovery of bacterial factors that impact the battle for nutrient metals at the host-pathogen interface. Understanding these adaptations may aid in the development of strategies to combat *S. aureus* infections, which remain a significant clinical challenge.

## MATERIALS AND METHODS

### Strains

All strains used in this study are listed in Table 1. Strains were cultured in tryptic soy broth (TSB) or on tryptic soy agar (TSA) plates at 37°C. Cultures grown in TSB were grown in 15 mL round-bottom polypropylene tubes with aeration lids at a 45° angle using an Innova44 incubator with shaking at 180 rpm.

**TABLE 1:** Strains used

Strain	Genotype	Description	Reference
Newman	WT	Wild-type, methicillin-sensitive clinical isolate	(60)
Newman	*fur::tetM*	Transduced from previously related mutant loci	(61)
Newman	∆*zur*	In-frame unmarked deletion of *zur* generated by allelic exchange	(62)
Newman	∆*mntR*	In-frame unmarked deletion of *mntR* generated by allelic exchange	(63)
Newman	∆*clpP*	Clean knockout	(64)
Newman	∆*clpP geh::clpP*	*clpP* stably integrated in L54A attB site within *geh*, contains *clpP* native promoter via pCL25	(64)

### Growth assays with calprotectin

Recombinant human calprotectin WT and the mutant of the His6 site (∆s1CP) were expressed and purified as described previously (7, 13). Overnight *S. aureus* cultures started from three independent colonies were subcultured at 1:50 into 5 mL of TSB for 1 h at 37°C in a shaking incubator. Back-diluted cultures were then inoculated at 1:100 into TSB containing 60% calprotectin buffer (20 mM Tris-HCl pH 7.5, 100 mM NaCl, 5 mM β-mercaptoethanol, and 3 mM CaCl_2_) supplemented with desired concentrations of recombinant human calprotectin. The concentrations of calprotectin used were 300 µg/mL WTCP and 600 µg/mL ∆s1CP. OD_600_ was measured at 30-min intervals over the course of 20 h at 37°C using an EPOCH two-plate reader (BioTek).

### RNA isolation

Cultures were grown as described above with a total volume of 5.5 mL. The cultures were treated with 300 µg/mL of WTCP, 600 µg/mL of ∆s1CP, or vehicle control (60:40 calprotectin buffer to TSB ratio). All samples were matched by growth phase and were collected for RNA isolation at mid-exponential growth phase. Differences in strain growth between the different conditions were taken into account and resulted in collection of samples between 4 and 7.5 h corresponding with an OD of 0.18–0.3, depending on the strain and treatment condition. All treatments were done in triplicate. Cultures were removed from the shaking incubator, centrifuged, and stored at −80°C until the time of RNA isolation as detailed in the supplemental material.

### RNA sequencing and data analysis

RNA sequencing was performed by Vanderbilt Technologies for Advanced Genomics Core Facility (VANTAGE) similarly to previously described (63). For more details, see the supplemental material.

### sRNA identification

RNAseq paired end fastq files were trimmed using trimmomatic v. 0.39 (65) parameters: LEADING and TRAILING were set to three with a MINLEN set to 36. Trimmed reads were checked for quality using FastQC v. 0.12.1 (https://www.bioinformatics.babraham.ac.uk/projects/fastqc). Trimmed reads were mapped to Newman reference ascension NC_009641.1 using Bowtie2 aligner (66). Samtools v. 1.9 (67) was used to generate sorted BAM files for the three condition sample replicates. Apero v. 1.0.3 (68) was used to identify the 5’ end of small targets, report read coverage, and annotate predicted transcripts through association with the closest coding sequence. Samples with sRNA hits in 2 out of 3 replicates were considered, and data for the third replicate was imputed when needed using a module for predictive mean matching in the R-based program mice (69). Due to the fact that multiple start sites and frequencies were identified for each sRNA region, the highest called frequency was used for each predicted transcript, utilizing a minimum 100× cutoff. Resulting putative sRNAs were visualized by screening mapped reads in CLC Workbench v. 22.0.1 to omit any regions that resembled UTRs and/or transcriptional readthrough. Finalized regions containing putative sRNAs were compared with the FPR3757 genome to extract known sRNA annotations (39). Frequencies for each sRNA were then used to calculate Log_2_ fold change for each condition compared with vehicle control.

### Transposon library generation

The vector pBURSA containing the *Bursa aurealis* minimariner transposable element and the pMG020 containing the *Himar* one transposase driven by the *lgt* promoter were used to generate the transposon mutant pool in the *S. aureus* Newman strain. The library was generated as previously described (70). For detailed methods, see the supplemental material.

### Tn-seq screen, library preparation, and analysis

A single aliquot of frozen *S. aureus* Newman Transposon library was inoculated at a ratio of 1:50 in 5 mL of TSB and grown for 1 h at 37°C with shaking at 180 rpm. An aliquot of this input library was also saved for sequencing. After 1 h of growth, the library was subcultured 1:100 into 5.5 mL aliquots of three different treatment conditions, vehicle control (60:40 calprotectin buffer to TSB ratio), 300 µg/mL of WTCP, or 600 µg/mL of Δs1CP. At 6 h, cultures were pelleted and stored at −80°C. This experiment was performed in triplicate.

DNA libraries from the experimental conditions were prepared for sequencing using the homopolymer tail-mediated ligation PCR (HTML-PCR) technique as previously described (71, 72). For more detailed methods, see the supplemental material.

### *S. aureus* mouse infections

All animal experiments were reviewed and approved by the Vanderbilt University Institutional Animal Care and Use Committee (Protocol number: M1900043). Procedures were performed according to the institutional policies, Animal Welfare Act, NIH guidelines, and American Veterinary Medical Association guidelines on euthanasia. C57Bl/6J female mice were purchased from Jackson Laboratories and infected at 6–8 weeks of age. The day prior to infection, single colonies of *S. aureus* were inoculated into TSB and grown for 16 h at 37°C with shaking at 180 rpm. Overnight cultures were subcultured into TSB and grown for 3 h. Bacteria were then centrifuged, washed three times, and resuspended in PBS. Female mice were retro-orbitally infected with 1 × 10^7^ *S. aureus* CFU in 100 µL of PBS. Infection proceeded for 96 h at which point mice were humanely euthanized and organs were harvested to determine bacterial burdens. Hearts, livers, spleen, and kidneys were homogenized in sterile PBS using a bullet blender tissue homogenizer (Next Advance), and serial dilutions were plated onto TSA to determine CFUs.

### Statistical analysis

Data were imported to GraphPad Prism for graphics generation and statistical analysis. Data were analyzed as indicated in figure legends. Asterisks indicate the statistical significance: *P < 0.05, **P < 0.01, ***P < 0.001, ***P < 0.0001, ns = not significant.

## Data Availability

Raw sequencing files obtained from RNA sequencing and transposon sequencing experiments are available in the National Center for Biotechnology Information (NCBI) Gene Expression Omnibus (GEO) under accession numbers GSE265954 and GSE266003.

## SUPPLEMENTAL MATERIAL

Supplemental figures mbio.01389-24-s0001.docx Fig. S1 and S2.

Supplemental text mbio.01389-24-s0002.docx Supplemental Materials and Methods.

Supplemental tables mbio.01389-24-s0003.xlsx Tables S1 to S11.

ASM does not own the copyrights to Supplemental Material that may be linked to, or accessed through, an article. The authors have granted ASM a non-exclusive, world-wide license to publish the Supplemental Material files. Please contact the corresponding author directly for reuse.
