# Supplementary material for: Coordinated adaptation of Staphylococcus aureus to calprotectin-dependent metal sequestration
Source: mBio. 2024 Jun 26;15(7):e01389-24. doi: 10.1128/mbio.01389-24 (PMC11253595; doi:10.1128/mbio.01389-24)
Supplement: Supplemental figures — Fig. S1 and S2. [file mbio.01389-24-s0001.docx]

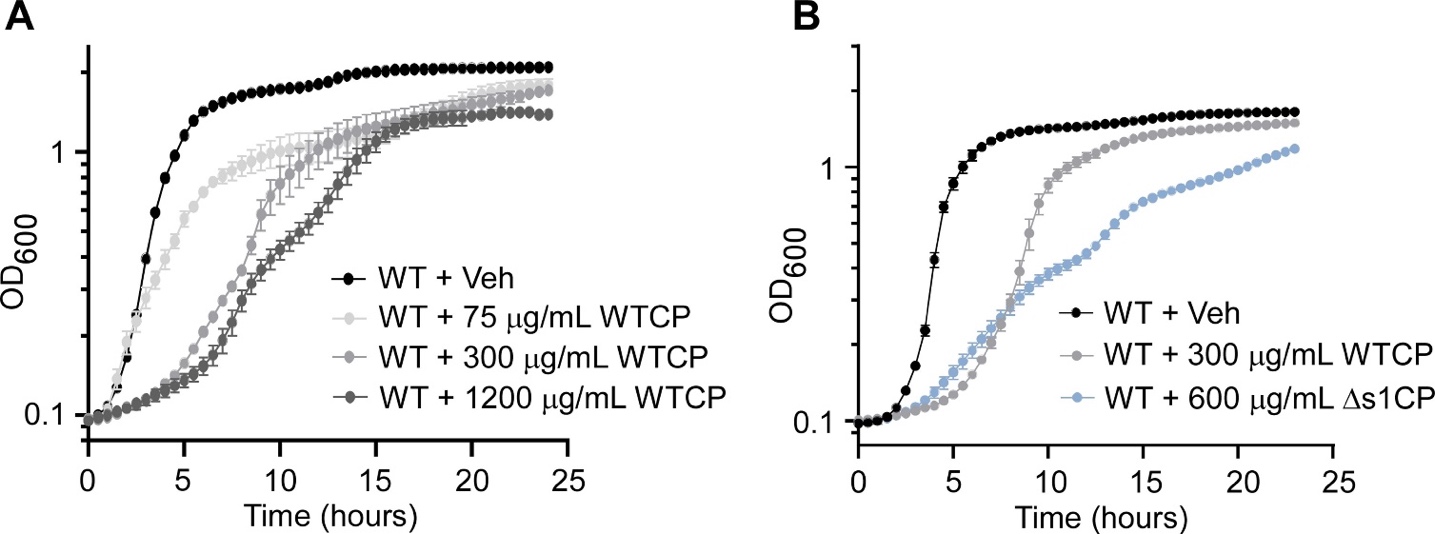


**Figure S1: Concentrations of calprotectin selected for these studies**. **(A)** Growth kinetics of *S. aureus* WT in vehicle and increasing concentrations of WTCP were monitored for 24h. **(B)** Growth kinetics of *S. aureus* WT in vehicle, 300 µg/mL WTCP, or 600 µg/mL ∆s1CP. Data shown are averages of 3-6 biological replicates done in triplicate (mean ± SEM).
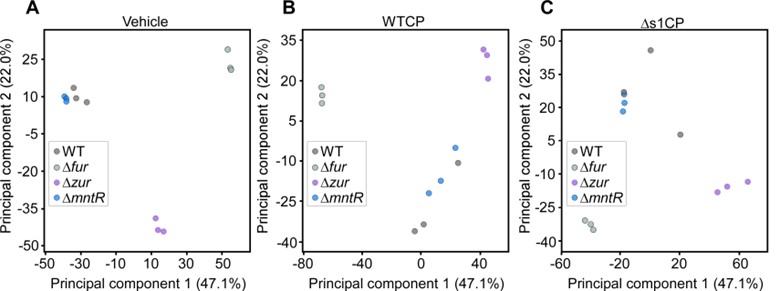


**Figure S2: PCA plots from RNA sequencing samples.** **(A)** PCA plots for vehicle control **(B)** PCA plots for samples treated with 300 µg/mL WTCP **(C)** PCA plots for samples treated with 600 µg/mL ∆s1CP.
