# Supplementary material for: Coordinated adaptation of Staphylococcus aureus to calprotectin-dependent metal sequestration
Source: mBio. 2024 Jun 26;15(7):e01389-24. doi: 10.1128/mbio.01389-24 (PMC11253595; doi:10.1128/mbio.01389-24)
Supplement: Supplemental text — Supplemental Materials and Methods. [file mbio.01389-24-s0002.docx]

**Supplementary Information**

**RNA isolation.** For the isolation of RNA, the QIAGEN RNeasy kit was used per recommendations from the manufacturer. Briefly, the bacterial pellets were resuspended in 100 µL of TE buffer. Resuspended bacteria were homogenized in a bead beater with Lysing Matrix B beads (MP Biomedical) at a speed of 6 m/s for 40 seconds. After, 650 μl of RLT buffer with 1% (v/v) β-mercaptoethanol was added and samples were homogenized again in a bead beater at a speed of 6 m/s for 40 seconds. Samples were centrifuged for 30 seconds to separate the beads and the upper phase was collected and mixed with 900 μL of ethanol. The mixed solution was then loaded onto the RNeasy column and subsequent isolation steps were performed using the QIAGEN RNeasy Kit following the protocol from the manufacturer. Genomic DNA was depleted from final RNA samples by treatment with TURBO DNA-free kit (Invitrogen).

**RNA sequencing and data analysis.** RNA sequencing was performed by Vanderbilt Technologies for Advanced Genomics Core Facility (VANTAGE). RNA quantity was determined using a Qubit Fluorometer (Thermofisher). A quality control analysis of the total RNA sample was performed using the Bioanalyzer TapeStation with the RNA 6000 Nano Kit (both Agilent). DNase-treated RNA with an RNA integrity number greater than 6 was used for ribosomal RNA depletion using RiboMinus Transcriptome Isolation kit (bacteria) and the RiboMinus Concentration Module (both Invitrogen). Library construction was performed using the NEBNext Ultra II RNA Library Prep kit for Illumina and NEBNext Multiplex Oligos for Illumina (both NEB). Sequencing of the constructed libraries was performed using NovaSeq 6000 instrument in combination with the NovaSeq 6000 S4 Reagent Kit (both Illumina) at Paired-End 150bp targeting an average of 50M reads per sample.

Bioinformatic analysis was performed using CLC Genomics Workbench Software. Paired-end sequencing data in fastq format were imported into CLC Genomics Workbench and trimmed to remove barcodes and adapter sequences. The remaining ribosomal RNA reads were depleted computationally by aligning all reads to a sequence list containing all rRNA sequences for the *Staphylococcus aureus* genome (NC_009641). Unmapped (non-rRNA) reads were collected and utilized for “RNA-seq analysis” function (standard settings, similarity fraction of 0.8) to generate expression values for each gene. A p-value of 0.05 and a max group mean greater than or equal to 10 was used as cutoff.

**Transposon library generation.** The vector pBURSA containing the *Bursa aurealis* minimariner transposable element and the pMG020 containing the *Himar* 1 transposase driven by the *lgt* promoter were used to generate the transposon mutant pool in the *S. aureus* Newman strain. The library construction was generated as previously described (1). Briefly, the pMG020 plasmid was transformed into RN4220 and a Φ-85 phage lysate was generated immediately for future steps. If more lysate was needed, pMG020 was freshly transformed into RN4220. *S. aureus* Newman strain containing pBursa was transduced with pMG020 phage lysate and after transduction multiple washes in TSB 0.5% sodium citrate were performed before plating on TSA 0.5% sodium citrate plates with 6 μg/mL of chloramphenicol and 1 μg/mL of tetracycline. Plates were incubated at 30°C for 36 hours and 6 individual colonies from the transduction were resuspended in PBS and plated on 150-mm plates containing TSB with 0.5% sodium citrate and 10 μg/mL erythromycin. Plates were incubated at 43°C for 48 hours to allow for transposition to occur. Library construction was repeated until approximately 100,000 individual colonies were collected. The pooled libraries were resuspended in TSB with 10 μg/mL erythromycin and 25% glycerol, vortexed, and stored at -80°C at a concentration of 1x10^10^ CFUs/ml. Spot plating of the library on TSA plates with or without antibiotics (10 μg/mL chloramphenicol, 10μg/mL erythromycin, or 10μg/mL tetracycline) confirmed curing of the plasmids pMG020 and pBursa in greater than 99% of bacterial cells. The library was confirmed to have approximately 80,000 independent transposon mutants as verified by Illumina sequencing analysis.

**Tn-seq screen, library preparation, and analysis.** DNA libraries from the experimental conditions were prepared for sequencing using the homopolymer tail-mediated ligation PCR (HTML-PCR) technique as previously described (2, 3). Modifications from this protocol involved the use of the KAPA HiFi HotStart DNA Polymerase (Roche) for the PCR cycles and the use of the transposon-specific primers olj510 and olj511 as previously described in Grosser et al. (1). In brief, genomic DNA (gDNA) from *S. aureus* cultures was extracted using the DNeasy Blood and Tissue Kit (QIAGEN). All DNA samples were then normalized to 100 ng/μL in ultra-pure water. Samples were then processed using the DTR gel filtration cartridges (Edge Biosystems Inc 42453) using the manufacturer’s recommendations. The sample (50 μL) was sheared in Covaris tubes using the Covaris LE220 instrument to generate 350bp fragments. Sheared DNA was treated with terminal deoxytransferase to generate a 3’ poly C-tail sequence, and two rounds of nested PCR were employed to amplify transposon junction regions. These products were multiplexed using 8-bp indexing primers and sequenced on the Illumina Hi-Seq 2500 at Tufts University Core Facility. Following sequencing, raw files were uploaded to the Galaxy Server from Tufts University to trim the reads, filter for quality, map to the *S. aureus* genome, and determine fitness using a “Dval” value for every gene in each library pool. A “Dval” score represents the aggregate number of reads for all transposon insertions within a gene in a given library sample, divided by the total number of predicted reads for that gene based on its size and the total number of reads obtained on the library pool. Dval scores in calprotectin treatments were normalized to Dval scores in vehicle control to calculate a fitness score for each gene in each experimental condition. Fitness scores were Log_2_ transformed and used to calculate a Z-score using the average and standard deviation of the population. A Z-score cutoff for significance was set at two standard deviations away from the mean.

**References**

1. Grosser MR, Paluscio E, Thurlow LR, Dillon MM, Cooper VS, Kawula TH, Richardson AR. 2018. Genetic requirements for *Staphylococcus aureus* nitric oxide resistance and virulence. PLoS Pathog 14:e1006907.

2. Lazinski DW, Camilli A. 2013. Homopolymer tail-mediated ligation PCR: a streamlined and highly efficient method for DNA cloning and library construction. Biotechniques 54:25-34.

3. van Opijnen T, Lazinski DW, Camilli A. 2015. Genome-Wide Fitness and Genetic Interactions Determined by Tn-seq, a High-Throughput Massively Parallel Sequencing Method for Microorganisms. Curr Protoc Microbiol 36:1E 3 1-1E 3 24.
